# Advances in the treatment of relapsed/refractory marginal zone lymphoma

**DOI:** 10.3389/fonc.2024.1327309

**Published:** 2024-01-25

**Authors:** Haotian Wang, Xin Wan, Ying Zhang, Jing Guo, Ou Bai

**Affiliations:** Department of Hematology, The First Hospital of Jilin University, Changchun, Jilin, China

**Keywords:** lymphoma, inert non-Hodgkin lymphoma, marginal zone lymphoma, targeted therapy, mechanism

## Abstract

Marginal zone lymphoma (MZL) is the second most common subtype of inert B-cell non-Hodgkin’s lymphoma, accounting for 5–15% of non-Hodgkin’s lymphoma cases. Patients with MZL have a long survival period, with a median survival of >10 years, and patients treated with a combination of anti-CD20 monoclonal antibody can achieve an overall effective rate of 81%. However, 20% of patients with MZL show relapse or experience disease progression within 2 years, with a median survival of only 3–5 years. Currently, the treatment options for patients with relapsed/refractory (R/R) MZL are limited, underscoring the pressing need for novel therapeutic drugs. The advent of novel anti-CD20 monoclonal antibodies, small molecule kinase inhibitors, immunomodulators, and other therapeutic strategies has ushered in a new era in the treatment of R/R MZL. Our objective is to summarize the existing treatment strategies, including immunotherapy and the emergent targeted therapies, and to evaluate their effectiveness and safety in the management of R/R MZL. By doing so, we aim to provide a clear understanding of the therapeutic landscape for R/R MZL, and to guide future research directions toward improving the prognosis and quality of life for patients afflicted with this challenging disease.

## 1 Introduction

Marginal zone lymphoma (MZL) originates in the follicular marginal zone and is a common subtype of inert non-Hodgkin lymphoma (iNHL). According to the latest World Health Organization (WHO) classification, MZL can be divided into extranodal MZL (EMZL), splenic MZL (SMZL), and nodale MZL (NMZL) (1). MZL accounts for approximately 5–15% of cases of non-Hodgkin’s lymphoma (NHL), EMZL for 70%, SMZL for 20%, and NMZL for 10% (2). The median age of MZL onset is approximately 67 years, depending on the subtype (3). No significant difference has been identified in the proportion of men and women affected; however, a female predominance is present in specific extranodal sites, such as the parotid and breast glands (4). The stomach is the most common extranodal site of MZL, followed by the adnexa of the eyes, lungs, skin, and salivary glands (2–4). The etiology of MZL is associated with chronic immune irritation caused by infectious factors. The pathogenic microorganisms that have been associated with the development of MZL include *Helicobacter pylori* (HP), *Chlamydia psittaci*, *Campylobacter jejuni*, *Borrelia burgdorferi*, and hepatitis C virus. HP infections are closely associated with gastric EMZL (5, 6). Immunoglobin heavy chain variable region gene rearrangements, chromosomal translocations and gene mutations are also involved in the development of MZL (7, 8). MZL progresses slowly and has a good prognosis, with a median survival of > 10 years (8). With the rising prevalence of MZL, immunochemotherapy has emerged as the go-to approach for its treatment. The forefront of this treatment strategy lies in the use of anti-CD20 monoclonal antibody, either as a standalone therapy or in conjunction with chemotherapy. This innovative combination has been proven to be the optimal first-line treatment option for MZL patients, providing them with a unique and effective approach to combat the disease. Specifically, these treatments, include rituximab combined with chlorambucil (R plus Chlorambucil); rituximab, cyclophosphamide, doxorubicin, vincristine, and prednisolone(R-CHOP); rituximab, cyclophosphamide, vincristine, and prednisolone (R-CVP); rituximab and bendamustine (BR), among others (9–14). Studies have revealed that the utilization of immunochemotherapy, specifically the anti-CD20 monoclonal antibody-based approach, has resulted in a remarkable 81% objective response rate (ORR) among patients (15). Furthermore, numerous research studies have indicated that the BR regimen exhibits superior efficacy and safety when compared to the R plus Chlorambucil and R-CVP regimens for the primary treatment of MZL (ORR 100% vs. 94.7% vs. 88%; complete remission [CR] 98% vs. 78.8% vs. 60%, respectively) (10–12) (**Table 1**).

**Table 1:** Efficacy and safety of first-line treatment for MZL.

Regimen	NO. of MZL	NO. of ORR(%)	NO. of CR/CRu(%)	Survival	AEs (grade ≥3)
R (10)	138	108 (78.3)	77 (55.8)	5-year PFS:72%	Infections 3%
R-Chlorambucil (10)	132	125 (94.7)	104 (78.8)	5-year PFS:57%	Neutropenia 14.4%; Lymphocytopenia 3.8%;Infections 3%
R-CVP (11)	40	35 (88)	24 (60)	3-year PFS and OS:59% and 95%	Neutropenia 10.5%;Febrile neutropenia 1.7%
BR (13, 14)	57	57 (100)	56 (98)	7-year EFS and PFS:87.7% and 92.8%	Lymphocytopenia 34%;Neutropenia 20%;Leucopenia 5%
BR (12)	237	221 (93.2)	192 (81)	5-year PFS and OS:80.5% and 89.6%	
BR/R-CVP/R-CHOP (15)	96	78 (81.3)	17 (17.7)	4-year PFS and OS:64.1% and 78.1%	Neutropenia 38.7%; Infusion-related reactions 11.8%;Febrile neutropenia Febrile neutropenia (9.7%)
BG/G-CVP/G-CHOP (15)	99	81 (81.8)	17 (17.2)	4-year PFS and OS:72.6% and 81.8%	Neutropenia 49.5%; Thrombocytopenia 11.9%; Pneumonia 10.9%

MZL, Marginal zone lymphoma; AEs, Adverse events; R, Rituximab; B, Bendamustine; CVP, Cyclophosphamide, vincristine, and prednisolone; CHOP, Cyclophosphamide, doxorubicin, vincristine, and prednisone; G, Obinutuzumab; ORR, Overall response rate; CR, Complete response; PFS, Progression-free survival; OS, Overall survival; EFS, Event-free survival.

While these treatments have significantly improved patient prognosis, several therapeutic challenges persist. Firstly, 20% of patients experience disease progression within 24 months of initial treatment (POD24), with a median survival of only 3-5 years (8). Secondly, 5-10% of MZL patients can transform into aggressive lymphomas, the mechanisms of which remain unknown. Histological transformation (HT) into aggressive lymphomas correlates with a poor prognosis in MZL; factors such as POD24, high-risk MALT International Prognostic Index (MALT-IPI) groups, and the detectability of monoclonal protein (m-protein) at diagnosis all increase the risk of HT (8, 16–18). Thirdly, high-risk MALT-IPI groups and detectable m-protein at diagnosis can also serve as indicators of poor prognosis in MZL (18, 19). For these patients with poor prognostic R/R MZL, there is an urgent need for new, effective, and safe treatment options to provide more therapeutic choices and prolong progression-free survival (PFS) in MZL patients. The development and application of novel anti-CD20 monoclonal antibodies, small molecule kinase inhibitors, immunomodulators, and other treatment strategies have ushered in a new era in the treatment of R/R MZL.

## 2 Methodology

In this review, we conducted a comprehensive literature search on studies published in recent years concerning the treatment of R/R MZL. This included searching for relevant research articles and clinical trial reports in databases such as PubMed, Embase, and the Cochrane Library. We integrated those studies that provided valuable information on treatment strategies, effectiveness, patient survival rates, and safety. We selected data from authoritative and reliable medical and scientific databases. Furthermore, we assessed the quality and risk of bias in the studies to ensure the scientific integrity and fairness of our review.

## 3 Current treatments

### 3.1 Novel anti-CD20 monoclonal antibodies

**Figure 1:**
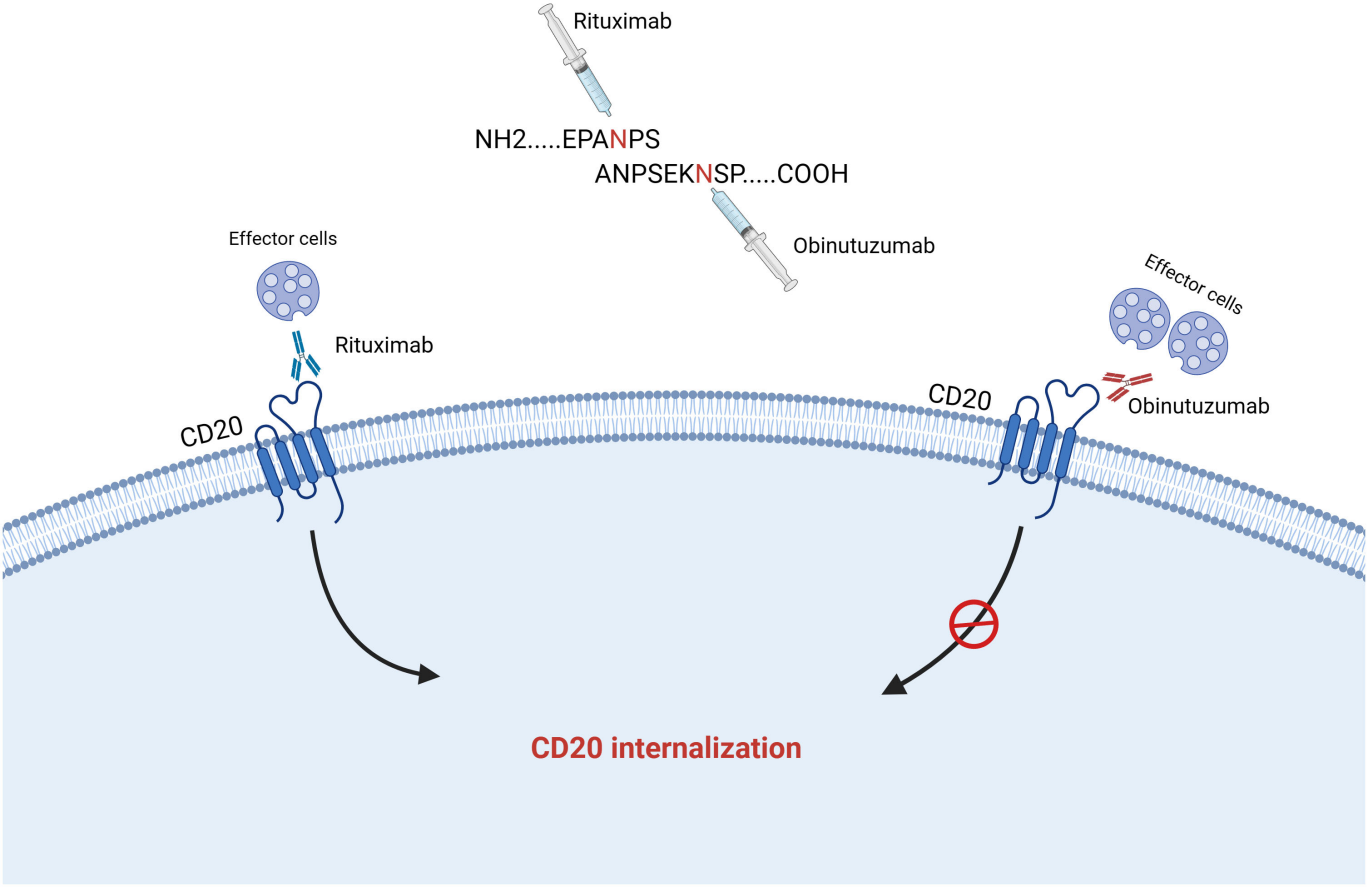
Difference in tumor-killing mechanism between obinutuzumab and rituximab.

Obinutuzumab (GA101; G) is the first humanized, glycosylation-modified type II CD20 monoclonal antibody. GA101 has a different sequence that binds to the CD20 receptor than the type I rituximab, binding mode and a different tumor-killing mechanism. The tumor-killing mechanism of GA101 is different from that of rituximab in the following ways (**Figure 1**): 1) GA101 has a different CD20 antibody-antigen recognition epitope than rituximab; GA101 has a unique CD20 antigen recognition epitope (170)ANPSEKNSP ^178^, which only partially overlaps with rituximab’s antigen recognition epitope ^168^EPANPS ^173^. When rituximab’s core antigen recognition epitope 171 has a mutation in the aspartic acid (N) position, its affinity decreases significantly, and it fails to recognize the CD20 antigen, whereas GA101’s core antigen is closer to the C-terminal end; thus, some rituximab-resistant patients can still benefit from GA101 (20, 21). 2) Rituximab incorporates into lipid rafts after binding to the CD20 antigen, resulting in fewer surface-recognizable targets. Low or absent CD20 expression is the cause of rituximab treatment resistance, whereas GA101 does not incorporates into lipid rafts after binding to the CD20 antigen and exerts a stronger direct cell death (DCD) effect (20–22). 3) The Fc segment of GA101 has been modified by glycosylation, which activates immune effector cells, exerting a stronger antibody-dependent cell-mediated cytotoxicity (ADCC) effect. GA101 induced a 35-fold enhancement of ADCC production compared with rituximab in an *in vitro* assay in human tumor cells (22, 23).

A randomized phase III study (GADOLIN), which included 194 patients with R/R iNHL treated with GA101 in combination with bendamustine (GB), including 28 patients with MZL, demonstrated that GB significantly prolonged PFS in patients with iNHL. The study followed up the patients for an average of 32 months, revealing a median PFS of 25.8 months in the overall intention-to-treat (ITT) group. Throughout the study 72.5% of patients in the GB group experienced grade ≥3 adverse events (AEs), which were most commonly neutropenia (34.8%), thrombocytopenia (10.8%), anemia (7.4%), and infusion reactions (9.3%) (24, 25).

### 3.2 Small molecule kinase inhibitors

Kinases are a class of phosphotransferases involved in substrate phosphorylation that transfers phosphate groups from high-energy donor molecules to specific target molecules (26). Protein kinases are the largest kinase group, acting on specific proteins to alter their activities, which, in turn, are involved in a range of cell signaling and regulatory processes (27). Mutations, translocations, dysregulation, and overexpression of protein kinases are intimately linked to the development of various ailments, including but not limited to tumors, inflammation, and autoimmune disorders (28). In recent years, small- molecule kinase inhibitors have attracted attention as promising targets for achieving anti-tumor effects by specifically blocking the signaling pathways necessary for tumor growth and proliferation and inhibiting tumor growth, metastasis, and recurrence (29). Small- molecule kinase inhibitors currently used to treat R/R MZL include Bruton’s tyrosine kinase (BTK) inhibitors (ibrutinib, zanubrutinib, acalabrutinib, and orelabrutinib) and phosphoinositide-3-kinase (PI3K) inhibitors (umbralisib) (30).

#### 3.2.1 BTK inhibitors

**Figure 2:**
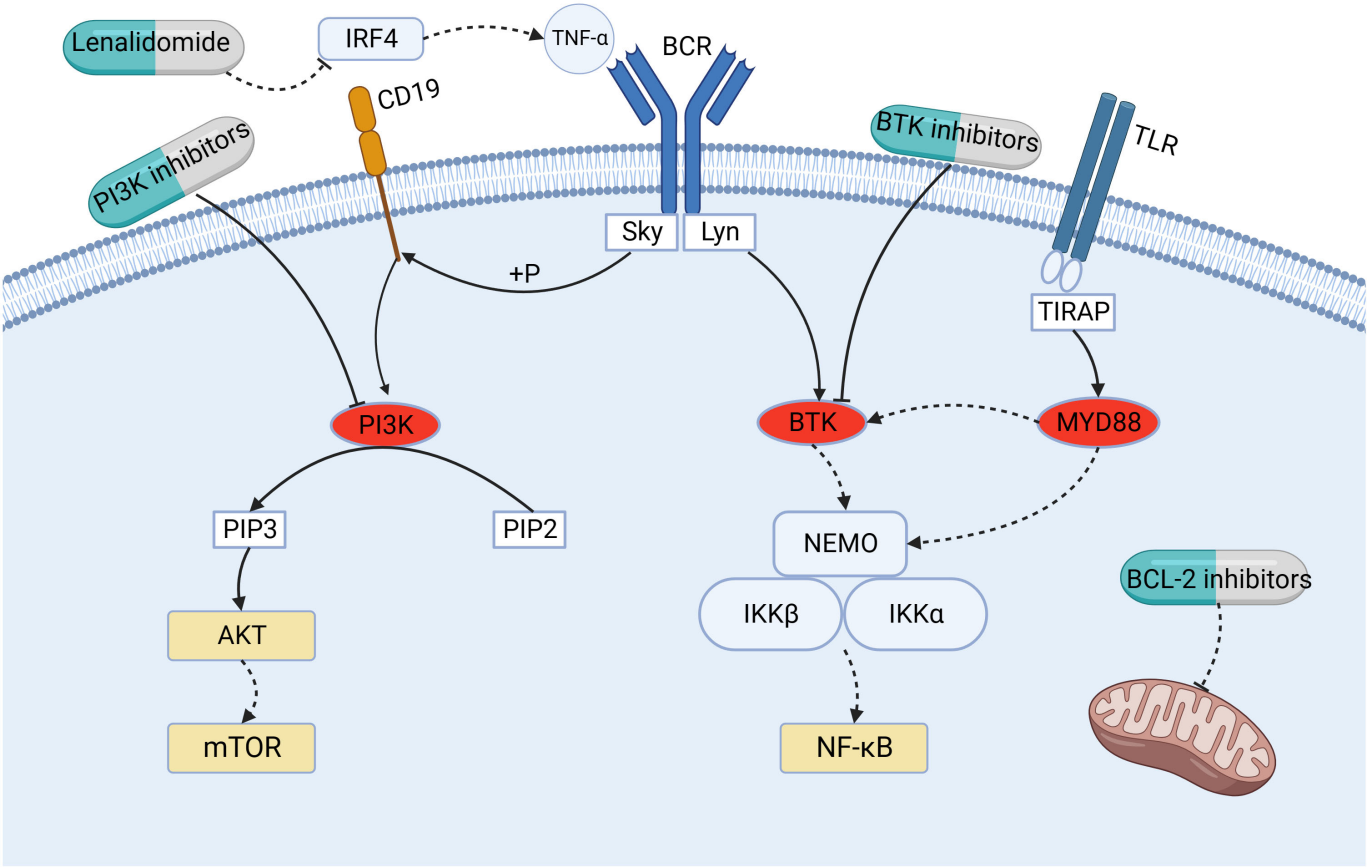
Mechanism of action of small- molecule kinase inhibitors (BTK inhibitors and PI3K inhibitors), lenalidomide, and BCL-2 inhibitors. BTK, Bruton’s tyrosine kinase; BCL-2, B-cell leukemia/lymphoma-2; BCR, B-cell antigen receptor; PI3K, phosphoinositide-3-kinase; IRF4, interferon-regulatory factor-4; MYD88, myeloid differentiation factor 88; NF-κ B, nuclear transcription factor-κ B; PIP2, phosphatidylinositol-4,5-bisphosphate; PIP3, phosphatidylinositol-3,4,5-trisphosphate; PKB/AKT, protein kinase B; mTOR, mammalian target of rapamycin; TLR, Toll-like receptor; TNF-α, tumor necrosis factor-α.

Under pathological conditions, persistent stimulation from microbes or self-antigens can cause over-activation of B-cell antigen receptor (BCR) signaling, leading to the proliferation of malignant B cells and promoting the development of MZL (31). BTK is a key kinase in the BCR signaling pathway, which participates and plays an important role in the regulation of B-cell proliferation, differentiation, and apoptosis (32). Studies have shown that in 7–15%,8%, and 6–7% of cases of SMZL, NMZL, and EMZL, respectively, mutations exist in the myeloid differentiation factor 88 (MYD88), an articulatory protein downstream of the Toll-like receptor (TLR). Specifically, the amino acid at position 256 in the sequence of MYD88 changes from leucine to proline when the base 794 in the coding sequence is mutated from T to C, and this missense mutation results in the MYD88 L256P mutant (33). The MYD88 L256P mutation can activate downstream interleukin 1 receptor-associated kinase 1 (IRAK1) and IRAK4 to further activate the nuclear transcription factor-κ B (NF-κ B) pathway, and it can simultaneously increase the activities of IRAK1 and IRAK4 kinases, which leads to the enhancement of NF-κ B pathway signaling (34, 35). Notably, the MYD88 L256P mutation can directly bind BTK to activate the NF-κ B pathway, and the binding of MYD88 to BTK causes BTK inhibitors to exert their antitumor effects (**Figure 2**).

The current clinical applications of BTK inhibitors for the treatment of R/R MZL include ibrutinib, zanubrutinib, acalabrutinib, and orelabrutinib. In a groundbreaking study (PCYC1121) (36), ibrutinib has shown remarkable efficacy in treating R/R MZL. This study included 63 patients with R/R MZL who had previously received rituximab or rituximab-based immunotherapy. The patients were administered 560mg/day of oral ibrutinib. After a median follow-up of 33.1 months, 60 patients were evaluable. The ORR was 58% and CR was 10%. The median duration of response (DOR) was 27.6 months, and the median PFS was 15.7 months. The median overall survival (OS) has not yet been reached. These findings suggest that ibrutinib monotherapy is effective in treating patients with R/R MZL who have previously received rituximab treatment, and can achieve a sustained remission. Based on this study, ibrutinib was approved in 2017 for patients with R/R MZL who received at least one CD20-containing monotherapy. In addition, the findings in a large multicenter retrospective study by Epperla et al. were consistent with those of the PCYC1121 study (37, 38).

Zanubrutinib is a highly selective second-generation irreversible BTK inhibitor. A phase II study (MAGNOLIA; BGB-311-214) (39, 40) enrolled 68 patients with R/R MZL and a median age of 70 years previously treated with at least one CD20-containing monoclonal antibody and zanubrutinib (160 mg orally two times/day). With a median follow-up of 27.4months, 66 patients with assessable efficacy had an ORR of 68.2% (45/66), CR of 25.8% (17/66), and partial remission (PR) of 42.4% (28/66). The 24-month DOR is 72.9%, and the 24-month PFS and OS are 70.9% and 85.9% respectively. In addition, zanubrutinib was well-tolerated, with no patients experiencing dose reductions due to AEs. Most of the AEs were grade 1 or 2. The most common AEs were diarrhea (22.1%), bruising (20.6%), and constipation (14.7%), which were manageable and reversible. Based on these results, zanubrutinib has received the green light from the United States Food and Drug Administration in 2021. This approval marks a significant milestone in the treatment of patients with R/R MZL who have undergone prior anti-CD20 monoclonal antibody-based therapy. Considering the incidence of cardiovascular events, Zanubrutinib has a better safety profile than Ibrutinib. Therefore, Zanubrutinib is recommended as the preferred option.

A phase II study (ACE-LY-003) (41) aimed to investigate the efficacy and safety of acalabrutinib monotherapy in patients with R/R MZL. A total of 43 R/R MZL patients received acalabrutinib monotherapy (100mg, orally twice daily). After a median follow-up of 13.3 months, 40 evaluable patients had an ORR of 52.2% (n=21) and a CR rate of 12.5% (n=5). The estimated median PFS was 27.4 months, while the median OS was not reached. The 12-month PFS and OS rates were 67.0% and 91.4% respectively. 40% (n=17) of patients experienced ≥ grade 3 treatment-emergent AEs (TEAEs), with the most common being neutropenia (14%), anemia (7%), and thrombocytopenia (5%). This study suggests that acalabrutinib monotherapy provides good efficacy and acceptable safety in patients with R/R MZL. However, due to the small sample size of the study, further evaluation of efficacy and safety is needed.

A multicenter, open-label, phase II study (ICP-CL-00104) (42) evaluated the effectiveness and safety of orelabrutinib in the treatment of R/R MZL. Ninety R/R MZL patients received orelabrutinib treatment (150mg, orally once daily). After a median follow-up of 23.4 months, the ORR was 58.9% (n=53), and the CR rate was 11.1% (n=10). The median PFS and OS were not reached, with 24-month PFS and OS rates of 75.8% and 86.8% respectively. Thirty-four patients (30.6%) reported ≥ grade 3 AEs, with the most common being neutropenia (8.1%), infectious pneumonia (6.3%), and anemia (4.5%). Based on the efficacy and safety demonstrated in this study, orelabrutinib was approved in China for the treatment of R/R MZL on April 23, 2023.

Overall, BTK inhibitors have demonstrated significant potential in the management of R/R MZL, providing durable responses and manageable safety profiles. However, further research is needed to directly compare the efficacy and safety of these drugs, and to better define their role in the treatment of R/R MZL.

#### 3.2.2 PI3K inhibitors

Phosphoinositide-3-kinase (PI3K) is a downstream effector of the BCR signaling pathway, which undergoes phosphorylation upon activation of BCR, triggering the conversion of phosphatidylinositol-4,5-bisphosphate (PIP2) to phosphatidylinositol-3,4,5-trisphosphate (PIP3), which in turn activates the protein kinase B (PKB/AKT)/mammalian target of rapamycin (mTOR) pathway (43–45). The PI3K family is divided into types I, II, and III, of which type I is involved in antigenic and co-stimulatory receptor signaling and is thought to be most closely related to tumor development (43). Type I PI3K is a heterodimer of a catalytic subunit (p110) and a regulatory subunit (p58). PI3K type I is divided into types IA and IB; type IA, depending on p110, can be further divided into PI3Kα, PI3Kβ, and PI3Kδ isoforms, and type IB is PI3Kγ. The PI3Kδ isoform, exclusively found in hematopoietic cells, exhibits significant expression in leukocytes, where it assumes a critical function in the growth, viability, and activity of B cells. When BCR overexpression leads to the continuous activation of PI3Kδ, the PI3K/AKT/mTOR pathway is activated to promote malignant lymphoma (43); molecularly targeted therapy against this pathway is currently a research hotspot (**Figure 2**).

Umbralisib, a groundbreaking compound, has emerged as a game-changing treatment option for patients with R/R iNHL. Combining the inhibitory effects on both PI3Kδ and casein kinase 1ϵ (CK1ϵ), this dual inhibitor has shown immense promise in a phase IIB trial (46). Focusing specifically on the MZL subgroup, the trial included 69 patients with an average age of 67 years and a median follow-up duration of 27.8 months. The outcomes were nothing short of extraordinary, with an ORR of 49.3% and a CR rate of 15.9%. The median PFS remained indeterminable, while the two-year PFS rate standed impressively at 50.5%. In terms of safety, 53.4% of patients experienced a grade ≥3 TEAE, with immune-related neutropenia (11.5%) and diarrhea (10.1%) being the most common. Of the included patients, 15.4% discontinued their medications because of TEAE. Therefore, the safety of PI3K inhibitors in the treatment of R/R MZL requires a high degree of attention.

These two aforementioned small-molecule kinase inhibitors are effective in the treatment of R/R MZL; however, the use of PI3K inhibitors is limited because of their toxic side effects. BTK inhibitors, with their favorable efficacy and safety profile, are the preferred new targeted agents for the treatment of R/R MZL.

### 3.3 Immunomodulators

In recent years, studies have shown that immunomodulators can improve the prognosis of patients with lymphoma, and the mechanism of their anti-tumor activity includes regulation of the microenvironment of tumor cells, anti-tumor cell angiogenesis, promotion of immune surveillance, regulation of immune activity, and promotion of tumor cell apoptosis (47–49). Lenalidomide is a 4-amino-glutaryl derivative of thalidomide, a member of a generation of immunomodulators, that has a strong immunomodulatory effect and a favorable safety profile (50). Lenalidomide, in its activation process, establishes a direct connection with the E3 ubiquitin ligase cereblon (CRBN) within myeloma cells (51). This interaction triggers swift ubiquitination and subsequent degradation of Aiolos and Ikaros, thereby reducing the levels of interferon-regulatory factor-4 (IRF4) in both transcription and protein forms (51). Additionally, it effectively inhibits the production of tumor necrosis factor-(TNF-), leading to the suppression of tumor cell proliferation (51) (**Figure 2**). In addition, lenalidomide inhibits IRF4 expression via CRBN, which subsequently causes BCR-dependent downregulation of NF-κ B (51). In a remarkable synergy, the combination of lenalidomide and rituximab (R2) unleashes a cascade of effects (52). Lenalidomide magnifies rituximab’s tumor-killing prowess by boosting CD16 expression, thereby empowering NK cells and monocytes to engage in antibody-dependent cellular cytotoxicity (ADCC). Additionally, it curbs angiogenesis, bolsters the NK cell population, and enhances rituximab-dependent NK cell-mediated cytotoxicity (48). This intricate interplay elevates the potential of R2 therapy to combat tumors.

Becnel et al. embarked on a phase II study (53), aiming to explore the effectiveness and safety of the revolutionary R2 regimen in treating stage III/IV MZL patients. Their pioneering research seeks to pave the way for a new standard of first-line treatment. A group of 30 individuals diagnosed with MZL, with an average age of 58 years, participated in a 28-day treatment cycle. The treatment involved taking 20 mg of lenalidomide orally for 21 days, from day 1 to day 21, and receiving a dose of 375 mg/m^2^ of rituximab on the first day. Over a span of 6-12 cycles, a total of 27 patients underwent assessment for a median follow-up period of 75.1 months. The results revealed an impressive ORR of 93%, with 70% of patients achieving CR. The median PFS stood at 59.8 months, showcasing the long-lasting impact of the treatment. Furthermore, the 5-year OS rate reached an outstanding 96%. Safety-wise, R2 was well-tolerated by patients with MZL, with most of the most common non-hematologic AEs being grade 1 or 2, a few being grade 3, and none being grade 4. Grade 3 hematologic AEs consisted mainly of neutropenia (33%) and leukopenia (7%), whereas grade 4 hematologic AEs included thrombocytopenia (3%) and leukopenia (3%). However, all AEs were manageable, and no patients developed serious infections.

In the MAGNIFY phase IIIB study that assessed the efficacy of the R2 regimen for R/R iNHL, 27 patients with MZL (22 evaluable) were enrolled, with an ORR of 55% and a CR of 45% (54). In addition, a phase III study (AUGMENT) by Leonard et al. comparing R2 versus rituximab monotherapy for R/R iNHL showed an ORR of 65%, CR of 29%, and median PFS of 20.2 months for the R2 treatment group in the MZL subgroup (55). These studies demonstrated that the R2 regimen has good efficacy in treating R/R MZL.

Based on the above studies, the R2 regimen demonstrated good efficacy and safety for treating both first-line and R/R MZL. It is now included in the Chinese Society of Clinical Oncology and National Comprehensive Cancer Network guidelines as a first- and second-line treatment option for patients with MZL.

## 4 Emerging therapies

### 4.1 BTK inhibitors

The first two generations of BTK inhibitors are covalent inhibitors. They exert their irreversible anti-tumor effects on BTK by binding to the cysteine residue at position 481 (C481). However, with long-term use, patients face the risk of mutation at the C481 site, where the cysteine residue can mutate to serine (C481S) (56, 57). This mutation changes the mode of interaction between the inhibitor and BTK from irreversible to reversible binding, thereby leading to drug resistance. Pirtobrutinib is a potent, non-covalent (reversible), highly selective inhibitor of both BTK and C481 mutant BTK. Pirtobrutinib functions by binding to BTK via an extensive network of interactions with water molecules in the ATP binding region, rather than directly interacting with the C481 site, demonstrating potential to overcome resistance to covalent BTK inhibitors (58, 59). On January 27, 2023, the FDA expedited approval of pirtobrutinib for the treatment of R/R MCL patients who have received at least two lines of systemic therapy, including BTK inhibitors (60). This is the first approved non-covalent (reversible) BTK inhibitor, and compared to previously approved BTK inhibitors, pirtobrutinib has over 300 times the selectivity for BTK (61).

In a study of pirtobrutinib for the treatment of R/R B-cell lymphomas, 36 patients were included in the MZL subgroup, of which 26 had previously received BTK inhibitor treatment (62, 63). The ORR was 50% (2.8% CR), and the ORR for MZL patients who had previously received BTK inhibitor treatment was 46.2%. With a median follow-up of 21.5 months, the median OS has not yet been reached, and the 24-month OS rate is 77.5%. The most common grade 3 or higher treatment-emergent adverse event (TEAE) was neutropenia (27.8%, n=10) and anemia (13.9%, n=5). No fatal TEAEs were observed.

The efficacy and safety profile demonstrated by pirtobrutinib appears promising. It could potentially serve as a viable treatment option for R/R MZL patients who have previously received covalent BTK inhibitors (cBTKis) treatment.

### 4.2 B-cell leukemia/lymphoma-2 inhibitors

The BCL-2 protein family plays a key role in mitochondria-mediated programmed cell death (**Figure 2**). Based on the presence of structurally conserved BH homologous structural domains, the BCL-2 family of proteins is divided into three major classes: anti-apoptotic proteins (BH1-4 structural domains), pro-apoptotic proteins with multiple BH structural domains (BH1-3 structural domains), and BH3-only pro-apoptotic proteins (64). The folded-loop structural domain (FLD) is a disordered region between BH3 and BH4, and lymphoma-specific mutations are mainly clustered in BH4 and FLD (64). Overexpression of the anti-apoptotic protein BCL-2 provides a survival advantage to malignant B cells and is involved in malignant B cell tumorigenesis, disease progression, and chemoresistance, making BCL-2 a potentially important therapeutic target for B -cell NHL.

In a phase I study by Davids et al. on the BCL-2 inhibitor venetoclax for the treatment of R/R iNHL, 106 patients (3 MZL) were enrolled and administered doses ranging from 200 mg to 1,200 mg daily, with an ORR of 67%, median follow-up period of 65 months, and median PFS and DOR of 21.2 months and 20.1 months, respectively, in the MZL group (65).

Sonrotoclax (BGB-11717) is a potent, highly selective new generation BCL-2 inhibitor, with a binding and inhibition potency more than ten times that of venetoclax. An ongoing Phase I/Ib study is exploring the efficacy and safety of sonrotoclax in R/R B-cell malignancies (66, 67). Among the 13 patients in the MZL subgroup, 12 were evaluable for efficacy, with an ORR of 67% (n=8), including a CR rate of 33% (n=4). The most common grade ≥3 TEAEs were neutropenia, febrile neutropenia/neutropenic sepsis, and tumor lysis syndrome (TLS) (n=2 [15%]). No TEAEs leading to death were reported.

Based on these findings, BCL-2 inhibitors provide new potential treatment methods for patients with R/R MZL. However, the current sample sizes of BCL-2 inhibitor-related clinical trials are small, and further cohort studies are needed.

### 4.3 Lysine-specific demethylase-1 inhibitors

LSD1 plays an important role in cell stemness, differentiation, cell motility, metabolic control, and epithelial-mesenchymal transition, which are closely related to tumor proliferation, invasion, metastasis, and poor prognosis (68). CC-90011, a potent, selective, reversible, and orally available inhibitor of LSD1, increase the expression of oncogenes and decreases the expression of pro-oncogenes, which, in turn, inhibits tumor cell proliferation (69). The recommended dose is 60 mg/week, with maximum tolerated and non-tolerated doses of 80 mg/week and 120 mg/week, respectively. Overall, 69 patients were enrolled in a phase I multicenter study (CC-90011-ST-001) (70) by Hollebecque et al, with 50 patients in the dose-escalation group and 19 in the dose-expansion group. In the dose-escalation group, only one patient with R/R MZL patient achieved CR, and the study is currently ongoing in anticipation of follow-up findings. Therefore, the effectiveness and safety of LSD1 as a new target for antitumor drugs in the treatment of lymphoma needs to be confirmed in further studies.

### 4.4 Chimeric antigen receptor T-cell therapy

CAR-T therapy employs advanced genetic engineering methods to equip T cells with CAR structures, enabling them to precisely target tumor antigens and effectively eliminate cancerous cells (71–73). To date, most clinical studies on CAR-T cell therapy for NHL have involved aggressive lymphomas, such as diffuse large B-cell lymphoma (DLBCL). The ZUMA-5 study (74) was the first to investigate the safety and efficacy of CAR-T therapy for the treatment of R/R iNHL in patients with R/R follicular lymphoma who received two or more prior therapeutic regimens (FL; grades 1-3a) or those with MZL. As of March 2023, a total of 31 patients with R/R MZL have been included in this study (75). With a median follow-up of 52.9 months, the ORR for all enrolled patients is 90% (70% CR). The median PFS in the MZL subgroup is 46.9 months, while the median OS has not yet been reached. Following three years of analysis among 152 treated patients (124 FL; 28 MZL), six severe AEs were reported. Of these, one was associated with the use of axicabtagene ciloleucel (axi-cel). With a median follow-up time of at least 4 years in the ZUMA-5 study, axi-cel demonstrated sustained durable responses and long-term survival in patients with R/R MZL.

### 4.5 Other therapies

CD3 × CD20 bispecific antibodies have demonstrated anti-B-cell NHL activity in recent clinical trials (76, 77). Recently, mosuntuzumab, epcoritamab, and glofitamab have been approved for the treatment of R/R B-cell NHL (78–80). An ongoing Phase III study on the treatment of R/R MZL using mosunetuzumab-lenalidomide (M-Len) holds the potential to transform the therapeutic approach for MZL patients, offering hope and possible clinical advantages (81). More clinical studies are expected to be conducted in the near future to confirm the efficacy and safety of the CD3 × CD20 bispecific antibody in R/R MZL.

## 5 Limitations

Firstly, our research primarily relies on previously published literature and data. This might result in the exclusion of the most recent research findings or ongoing clinical trial data. Additionally, due to language barriers, we may not be able to access all non-English studies. Secondly, our research may possess selection bias. Despite our efforts to choose the most relevant and valuable studies, some may have been excluded for various reasons, such as low quality of the study or significant discrepancies in the results compared to other studies. Lastly, there may be interpretation bias in our research. While we strive to remain objective and impartial, our subjective judgement in interpreting and analyzing data may influence the results to some extent. We acknowledge these limitations and will endeavor to avoid these issues in future research to provide more accurate and comprehensive information.

## 6 Conclusion

In summary (**Table 2**, **3**), while significant advancements have been made in the treatment of R/R MZL, certain limitations persist. The heterogeneity of R/R MZL adds an additional layer of complexity to its treatment, as the therapeutic approach must be tailored to the individual characteristics of the patient and the subtype of the disease. In recent years, the exploration of “chemotherapy-free” treatment modalities in the field of lymphoma has led to extensive trials of non-chemotherapy regimens based on small molecule targeted drugs in the treatment of indolent lymphomas. The combination of targeted drugs has emerged as a focal point of research, such as the combination of BTK inhibitors and anti-CD20 monoclonal antibodies. There is a pressing need for more comprehensive comparative studies to elucidate the optimal treatment sequence and combination in the management of R/R MZL.

**Table 2:** Efficacy and safety of current treatments for R/R MZL.

	Regimen	NO. of MZL	NO. of ORR (%)	NO. of CR/CRu (%)	Survival	AEs (grade ≥3)
SNovel anti-CD20 monoclonal antibodies	GB (24, 25)				median PFS: 25.8 months	Neutropenia 34.8%; Thrombocytopenia 10.8%; Anemia 7.4%; Infusion reactions 9.3%
BTK inhibitors	Ibrutinib (36)	60	35 (58)	6 (10)	median DOR and PFS: 27.6 and 15.7 months	Infections 22%; Anemia 16%; Major hemorrhage 3%
	Zanubrutinib (39, 40)	66	45 (68.2)	17 (25.8)	24-month PFS and OS: 70.9% and 85.9%	Infections 16.2%; Neutropenia 10.3%;thrombocytopenia 4.4%
	Acalabrutinib (41)	40	21 (52.5)	5 (12.5)	12-month PFS and OS: 67% and 91.4%	Neutropenia 14%; Anemia 7%; Thrombocytopenia 4.7%
	Orelabrutinib (42)	90	53 (58.9)	10 (11.1)	24-month PFS and OS: 75.8% and 86.8%	Neutropenia (8.1%); Infectious pneumonia (6.3%); Anemia (4.5%)
PI3K inhibitors	Umbralisib (46)	69	34 (49.3)	11 (15.9)	2-year PFS: 50.5%	Neutropenia 11.5%; Diarrhea 10.1%; ALT/AST increased 6.7%/7.2%
Immunomodulators	R2 (54)	22	12 (55)	10 (45)		Neutropenia 33%;Thrombocytopenia 11%
	R2 (55)	31	20 (65)	9 (29)	median PFS: 20.2 months	Neutropenia 50%;Leucopenia 7%; Anemia 5%

Relapsed/Refractory; MZL, Marginal zone lymphoma; AEs, Adverse events; BTK, Bruton’s tyrosine kinase; PI3K, phosphoinositide-3-kinase; GB, Obinutuzumab, bendamustine; R2, Rituximab, lenalidomide; ORR, Overall response rate; CR, Complete response; PFS, Progression-free survival; OS, Overall survival; DOR, Duration of response; ALT/AST, Alaninetransaminase/Aspartate transaminase.

**Table 3:** Efficacy and safety of emerging therapies for R/R MZL.

	Regimen	NO. of MZL	NO. of ORR(%)	NO. of CR/CRu(%)	Survival	AEs (grade ≥3)
BTK inhibitors	Pirtobrutinib (62, 63)	36	18 (50)	1 (2.8)	24-month OS: 77.5%	Neutropenia (27.8%); Anemia (13.9%)
BCL-2 inhibitors	Venetoclax (65)	3	2 (67)	0 (0)	median PFS and DOR: 21.2 and 20.1 months	
	Sonrotoclax (66, 67)	12	8 (67)	4 (33)		Neutropenia (15%); Febrile neutropenia/ neutropenic sepsis (15%); TLS (15%)
LSD1 inhibitors	CC-90011 (70)	1	1 (100)	1 (100)		
CAR-T	CAR-T (74, 75)	31			median PFS: 46.9 months; 48-month OS: 68%	Neutropenia 34%; Anemia 22%

R/R, Relapsed/Refractory; MZL, Marginal zone lymphoma; AEs, Adverse events; BTK, Bruton’s tyrosine kinase; BCL-2, B-cell leukemia/lymphoma-2; CAR-T, Chimeric antigen receptor T-cell; ORR, Overall response rate; CR, Complete response; PFS, Progression-free survival; OS, Overall survival; DOR, Duration of response.

In addition, it’s worth exploring whether advancements in the treatment of other types of lymphoma could inform the therapeutic approach for MZL. For instance, diffuse large B-cell lymphoma (DLBCL) shares a common origin with MZL as they are both malignant B-cell tumors. It’s noteworthy that 5-10% of MZL can undergo transformation (HT-MZL) (8). Studies have confirmed that compared to MZL, HT-MZL is characterized by elevated serum lactate dehydrogenase levels; upregulated expression of BCL-6, MUM-1, C-MYC, and Ki-67; more frequent mutations in TBL1XR1, CCND3, and ID3; and more common bone marrow involvement (82). These distinct features could provide valuable insights for the development of targeted therapies for MZL. In recent years, advancements in genomics and molecular biology have revealed numerous molecular similarities between MZL and DLBCL. These similarities primarily manifest in shared gene mutations, epigenetic modifications, and dysregulation of signaling pathways. Initial studies have identified shared mutations in key genes such as NOTCH2 and BCL2 in both MZL and DLBCL (83, 84). These genes play crucial roles in cell proliferation and differentiation, and their mutations can contribute to lymphomagenesis. Additionally, similar patterns of epigenetic modifications, such as DNA methylation and histone modifications, have been observed in both types of lymphoma, underscoring their significant roles in these diseases (85, 86). Lastly, there is indeed a convergence in the dysregulation of signaling pathways in MZL and DLBCL. This convergence is reflected in the inhibition or activation of key genes in these pathways, such as MYD88, CARD11, and TNFAIP3 (84, 87). The aberrations in these genes can lead to abnormal activation of the NF-κB and BCR signaling pathways, thereby contributing to the development of MZL and DLBCL. By delving deeper into the molecular intersections between MZL and DLBCL, we can not only gain a better understanding of the pathogenesis of these two types of lymphoma but also identify potential new therapeutic targets. We hope that further research in the future will provide more assistance in the diagnosis and treatment of MZL.

Addressing these limitations requires ongoing research and clinical trials to refine the treatment strategies for R/R MZL and improve patient prognosis.

## Author contributions

HW: Writing – original draft. XW: Writing – review & editing. YZ: Writing – review & editing. JG: Writing – review & editing. OB: Funding acquisition, Writing – review & editing.
